# Nicotine Dependence and Loss of Autonomy among Greek Adolescent Smokers: A Countrywide Cross-Sectional Study

**DOI:** 10.3390/ijerph17218191

**Published:** 2020-11-05

**Authors:** Soteris Soteriades, Athanasios Basagiannis, Elpidoforos S. Soteriades, Anastasia Barbouni, Varvara Mouchtouri, George Rachiotis, Christos Hadjichristodoulou

**Affiliations:** 1 Laboratory of Hygiene and Epidemiology, Faculty of Medicine, University of Thessaly, 41222 Larissa, Greece; ssotiriadis@uth.gr (S.S.); athbasagiannis@gmail.com (A.B.); mouchtourib@med.uth.gr (V.M.); grach@med.uth.gr (G.R.); 2 Healthcare Management Program, Faculty of Economics and Management, Open University of Cyprus, Nicosia 2210, Cyprus; esoteria@hsph.harvard.edu; 3 Environmental and Occupational Medicine and Epidemiology (EOME), Department of Environmental Health, Harvard T.H. Chan School of Public Health, Boston, MA 02115, USA; 4 Department of Public and Administrative Hygiene, National School of Public Health, 11521 Athens, Greece; abarbouni@uniwa.gr

**Keywords:** HONC, adolescent, cigarette, tobacco, smoking, nicotine dependence, loss of autonomy, Greece

## Abstract

Nicotine dependence is one of the main reasons for the continuation of smoking among adolescents. Loss of autonomy (LOA) is a measure of dependence. This study is the first to investigate LOA and its determinants among Greek adolescents. In 2013, 13-to-15-year-old middle-school students were selected by multi-stage clustered sampling. LOA was evaluated with the Hooked-on-Nicotine Checklist (HONC). Multiple univariate analysis was used to assess the association between adolescent demographics, smoking habits, and loss of autonomy. Three-hundred thirty-nine current smokers responded to the questionnaire (response rate: 82.3%). Of these respondents, 51.2% were male and 88.8% reported at least one LOA symptom. The mean HONC score was 4.13/10 (95% CI: 3.82–4.45). Higher scores were negatively associated with lower smoking frequency (cumulative odds ratio (cOR): 0.240, 95% CI: 0.144–0.400) and positively associated with lower age at first cigarette (cOR: 2.29, 95% CI: 1.38–3.82). Female gender was significantly associated with the prevalence but not the degree of LOA. Overall, the prevalence and the degree of nicotine dependence among adolescent smokers in Greece is similar to other countries. Frequent smoking and initiation of smoking at a younger age are linked to nicotine dependence, although it was not possible to make causal inferences. The relationship between nicotine dependence and gender remains unclear.

## 1. Introduction

Smoking constitutes the single most preventable cause of disability and death [1]. It is also the single most significant cause of cancer, and lung cancer in particular, in the general population [2]. In addition, smoking is one of the most significant causes of pulmonary problems including asthma and chronic obstructive pulmonary disease [3]. It is strongly associated with the development of cardiovascular diseases [4] and has been reported as an associated cause of several other health problems in both genders and for all ages [5,6,7].

One of the most disturbing findings of smoking-related research around the globe is the gradual decrease in the age of smoking initiation, reported in children as young as 7 years old, despite strong societal efforts to limit advertisement and marketing of smoking products to underage children [8,9]. Several studies have shown that the lower the age of smoking initiation among children, the higher the risk of becoming a regular smoker in adolescence and adulthood, the higher the risk of being a heavier smoker, and the harder for a smoker to quit [10,11]. Certain studies have also found that the age of smoking initiation is associated with genetic factors [12,13]. Along with the above findings, research shows that experimentation with cigarettes in young children leads to the development of nicotine addiction at an early stage and subsequent difficulty in quitting [14,15,16,17,18].

At this point, it is pertinent to the topic to clarify the distinction between addiction and dependence. Nicotine addiction is a behavioural disorder that involves the compulsive exposure to nicotine, due to its rewarding brain stimuli, despite its adverse consequences. Nicotine dependence, also known as withdrawal syndrome, is a state in which the individual develops unpleasant symptoms upon cessation of repeated nicotine use. Addiction and dependence may exist independently of one another (more rarely) or may occur simultaneously (more often). In both cases, the individual may find it difficult to quit smoking despite his intention to do so. Dependence may be characterised by loss of autonomy (LOA) [19,20,21]. Loss of autonomy is a situation that is conceptualised as loss of free will over quitting tobacco and it develops when the consequences of smoking (biological, psychological, behavioural) act as a barrier to quitting. The autonomy model allows for several independent mechanisms to contribute to the same clinical syndrome of dependence [19].

Based on the above perspective, studies have shown that even intermittent or occasional smoking in adolescents may develop nicotine dependence and LOA, as assessed by validated questionnaires including the Hooked-on-Nicotine Checklist (HONC) [22,23]. Endorsing a single item on the checklist indicates some degree of LOA, which increases as more items are endorsed. Specifically, adolescents who smoke even fewer than one cigarette per month have reported symptoms of LOA [24].

The HONC questionnaire was distributed in Greece in parallel with the Global Youth Tobacco Survey (GYTS), which is a standardised smoking-related survey that has been used worldwide to monitor smoking prevalence and associated risk factors among adolescents. Greece has one of the highest prevalence of adult smokers in Europe and globally [25], while GYTS surveys conducted in Greece have shown that the prevalence of smoking and the prevalence of use of other nicotine products among adolescents were also quite high [26,27].

The aims of the current study were the following: (a) to determine if different groups of Greek adolescent smokers have different degrees of LOA, and (b) to determine if there is a different prevalence of LOA (defined as a HONC score of one or more) among different groups of Greek adolescent smokers. These groups were distinguished based on age, sex, age at first cigarette, smoking frequency, and the presence of other smokers in the family. The study’s focus was on students’ use of conventional cigarettes only.

## 2. Materials and Methods

### 2.1. Study Population

The survey was conducted in Greece across the whole country in 2013. The study population consisted of middle-school students aged 13 to 15-years-old attending public or private schools. Details about the sampling methodology of the GYTS study can be found elsewhere [28]. Within the framework of this study, loss of autonomy was investigated by focusing specifically on students who reported having smoked conventional cigarettes at least once during the last 30 days (defined as current smokers). Participants were included in the analysis only if they claimed to be current smokers of conventional tobacco cigarettes. The use of other nicotine products, such as pipes, cigars, and electronic cigarettes, was not part of the inclusion or exclusion criteria.

### 2.2. Data Collection

Data were collected using a school-based, self-administered, pencil-and-paper survey, which included several questions on students’ demographics and their families, questions on tobacco smoking, and the use of other nicotine products. The questionnaire made a clear distinction between various categories of nicotine products: tobacco cigarettes, other combustible tobacco products (pipes, cigars), non-combustible products (chewed tobacco), and electronic cigarettes (the questionnaire asked specifically about electronic cigarettes but not about other electronic nicotine delivery systems). Furthermore, it contained questions pertaining to exposure to tobacco advertising, awareness of the health effects of tobacco, and nicotine addiction and dependence.

### 2.3. Predictor Variables

All variables were derived from questionnaire items and, in most cases, converted to binary variables. The following variables were used: age, gender, age at smoking initiation, the number of cigarettes smoked per day during the last month, and presence of other smokers in the family. Age had three categories: 13, 14, and 15-years-old. Number of cigarettes smoked per day was dichotomised: five cigarettes or less, or more than five cigarettes. Age at smoking initiation was also dichotomised: 11 or younger, or 12 or older. A question about smoking in the family asked about smoking by each parent and by siblings (multiple-choice question).

### 2.4. Outcome

Loss of autonomy (LOA), derived from the Hooked-on-Nicotine Checklist (HONC), was used as the study outcome. The questionnaire consists of 10 closed-type questions evaluating LOA in association with smoking. The HONC score was equal to the number of ‘yes’ responses to the 10 questions. The HONC score, denoting LOA, was used as a continuous variable (0–10) and was also converted into a dichotomous (0, ≥1) and a trichotomous variable (0 vs. 1–5 vs. 6–10). The dichotomous outcome variable was used mainly for comparison with other studies, which defined LOA as a positive HONC score. However, the trichotomous variable was hypothesised to convey more clinically relevant information.

### 2.5. Statistical Analysis

Prior to any statistical analysis, a weighting factor was applied to reflect the probability of sampling of each student. The weight calculation has been reported elsewhere [28]. Individual weights were scaled down so that the size of the sample would remain the same for the purposes of the statistical analysis. After applying the weights, a frequency analysis was performed.

Three univariate models were developed. For the first model, which used the continuous HONC score as the outcome, it was determined that the outcome variable did not follow a normal distribution; therefore, the appropriate non-parametric test (Mann–Whitney U or Kruskal–Wallis) was used for each simple univariate analysis. However, the multiple univariate analysis, to be developed using factorial analysis of variance (ANOVA), was abandoned since the outcome variable did not follow a normal distribution. For the second model, the trichotomous HONC score was used as the outcome. Pearson’s chi-square t-test was used for all simple univariate analyses. For the multiple univariate analysis, multiple ordinal regression was used in order to account for potential confounders. Finally, the third model used a binary outcome variable (HONC = 0 vs HONC ≥ 1). Potential interaction was investigated using an EVW hierarchical model, but none of the interactions were statistically significant and were, thus, excluded. Initially, all aforementioned predictor variables were included in each model and justification was given wherever a predictor was excluded.

All statistical tests were performed using a two-tailed significance level of 0.05. Point estimates were accompanied by 95% confidence intervals (95% CI). Missing values were ignored. IBM^®^ SPSS^®^ Statistics for Windows, Version 25.0 (IBM Corp., Armonk, NY, USA), was used for all statistical tests.

### 2.6. Ethical Approval

Participating students provided verbal consent and their parents were informed by mail. The Ministry of Education and Religious Affairs and the Institutional Review Board of the National School of Public Health (NSPH), located in Athens, Greece, approved the protocol of the study (ID: 41996/Γ2/6-4-2011 and 41996/Γ2/31-5-2012).

## 3. Results

**Figure 1:**
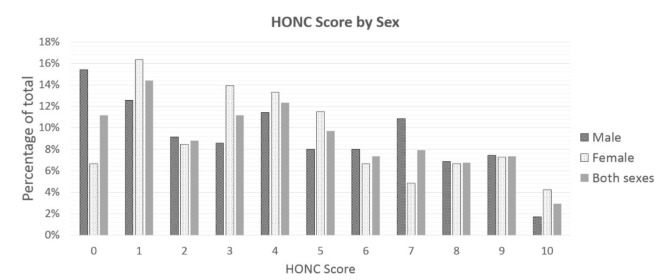
Distribution of Hooked-on-Nicotine Checklist scores by sex.

A total of 4096 students aged 13–15-years-old responded to the overall survey, of which 412 were current smokers, i.e., they reported having smoked at least once in the last 30 days (prevalence: 10.1%). Among this group, 339 students responded to the HONC questionnaire (response rate: 82.3%) and were included in the analysis. About 15.8% of HONC respondents were 13 years old, 37.6% were 14 years old, and 46.6% were 15 years old. A total of 51.2% were male and 88.8% reported at least 1 symptom of LOA based on HONC. Nearly all participants reported that other members of their family smoked (99.8%); therefore, this variable was excluded from any further analysis due to its low variability. The mean HONC score was 4.13/10 (95% CI: 3.82–4.45). The distribution of the predictor variables is presented in Table 1, while the distribution of HONC scores is presented (for both sexes together and separately) in Figure 1.

In Table 2, the results of the simple univariate analysis are presented for the quantitative outcome (described by the median) and the trichotomous categorical outcome (described by proportions). A significant difference between the median values of the continuous HONC score was found when comparing those who smoked five or less vs. more than five cigarettes per day, with the latter group reporting higher scores. The same was found when comparing those that initiated smoking at age 11 or younger as compared to those at age 12 or older, with the prior group reporting higher scores. The same variables were significantly associated with the HONC score when it was converted into a trichotomous variable.

Table 3 contains the results of the ordinal regression, which investigated the association between the trichotomous HONC score (0 vs. 1–5 vs. 6–10) and two predictors: smoking frequency and age of smoking initiation. The cumulative odds ratio (cOR) of scoring a higher HONC score for those who smoked less compared to those who smoked more frequently was 0.240 (95% CI: 0.144–0.400), which suggests a positive association between higher smoking frequency and higher LOA. On the other hand, the cOR of scoring a higher HONC score for those who started smoking at or below 11 years old was 2.29 (95% CI: 1.38–3.82), compared to those who started smoking at or above 12 years old, which suggests a positive association between lower age of smoking initiation and higher LOA. Both associations were statistically significant. The inclusion of age and sex in the ordinal regression model was also explored; however, it was decided to omit these two variables. The main reason was that their inclusion resulted in a violation of the assumption of proportional odds; in addition to this, their association with the outcome was non-significant.

A separate multiple binary logistic regression was carried out with a dichotomous HONC score (0 vs. ≥ 1). The detailed results can be found in the Supplementary Materials. In summary, it was found that female gender (adjusted odds ratio (aOR): 2.54, 95% CI: 1.18–5.43) and daily smoking frequency (aOR: 6.34, 95% CI: 1.61–25.1) were significantly associated with a positive HONC score, while the student’s current age and the student’s age at smoking initiation were not.

Finally, in order to check for bias caused by attrition, we investigated whether there were significant differences between those smokers who responded to the HONC and those who did not. These are the results can be seen in Table 4. It was found that the smokers who responded to HONC did not differ from smokers who did not respond to HONC in the distribution of age, gender, or their age at smoking onset. It was found, however, that smokers who did not respond to HONC where more likely to smoke fewer cigarettes per day.

## 4. Discussion

### 4.1. Interpretation of Results and Comparison with Other Studies

This is the first study to examine the association between the extent of smoking and loss of autonomy (LOA) among adolescents in Greece. It was found that a relatively high prevalence of LOA exists among 13–15-year-old smoking adolescents in Greece (88.8%). The biological factors, including genetic, pharmacological, and other homeostatic factors, influencing loss of autonomy should not be overlooked, although they were not the focus of this study. The psychological factors that affect LOA have become apparent through this questionnaire: negative symptoms like irritability, lack of concentration, urges and cravings, nervousness, restlessness, and anxiety. Social and cultural factors, such as smoking by other family members, which is abundantly common in Greece, peer pressure by friends, and the influence of role models also potentially contribute, especially in Greece where adult smoking rates are very high and where smoking was, at the time, ubiquitous in both public and private settings.

The mean HONC score of 4.13/10 indicates that the degree of LOA is similar to that reported for current adolescent smokers in other countries. In Romania [29], for example, in a group of 14–16-year-old students, the HONC score was 3.4–3.5/9 (which is equal to 3.8–3.9/10). On the other hand, the degree of LOA was found to be lower in one cross-sectional study from Florida, USA [30], among 14–18-year-old students, with a mean HONC score of 2.8/10, as well as among 13–17-year-old students in Malaysia [31], with a mean HONC score of 2.7/10; however, the latter survey also included exclusive users of electronic cigarettes (EC). With the exclusion of EC-exclusive users, the prevalence of LOA increased from 78.7% to 90.6%, which is similar to the observed prevalence in the current study. A study in New Zealand [32] also found a similar prevalence of LOA among 13–17-year-old students (87.9%), while the mean HONC score was slightly higher (4.9/10, SD: 3.3). A study among 14–17-year-olds in France [33] found a much higher mean score (5.5/10) and a higher prevalence of LOA (93.8%), but this was expected since it was carried out among daily smokers, not current smokers.

Two interesting findings, consistent with the results of other studies [19,32], are that students who began to smoke at a younger age and students who smoked more frequently had significantly higher degrees of LOA. The prior finding raises further concerns about the effects of nicotine on the child and adolescent brain. The results of this and other studies [34] point to the possibility that physical or mental dependence becomes more hard-wired and more permanent if the brain is exposed to addictive substances at an earlier developmental stage. The power of habit itself, whether addictive or not, must also not be understated: the earlier a habit is adopted, the harder it is to overcome. An important factor not explored in most of these studies is the use of other nicotine products. Furthermore, the integration of genetic factors into a broader model of nicotine dependence would improve our understanding of the problem, as noted in the introduction, although it was not explored by this study. If any reliable conclusions are to be drawn about youth nicotine dependence at the population level, total nicotine intake would be pertinent to this matter since conventional cigarettes are only one of many sources of nicotine. As for the association between higher smoking frequency and higher LOA, it is not possible to draw any inferences regarding the direction of causality since this is a cross-sectional study. It appears equally plausible that increased smoking frequency can aggravate nicotine dependence and that nicotine dependence can lead to increased smoking frequency.

Furthermore, it was possible to replicate the finding of other studies that girls have a significantly higher prevalence of LOA (HONC ≥ 1) compared to boys despite having lower levels of smoking, which is a repeated finding across different countries and cultures [19,20,24,35]. However, it is noteworthy that the association between female gender and LOA was not significant when dividing the HONC score into three categories. Indeed, it is apparent from Figure 1 that there is a big difference between boys and girls at HONC = 0, but this does not remain consistent across the spectrum of HONC scores. As seen in Table 2, there are more boys than girls in the ‘no dependence’ category but there are also more boys than girls in the ‘strong dependence’ category. Girls are more abundant in the ‘weak dependence’ category. Therefore, it seems that boys are more likely to occupy the ends of the spectrum, while girls are more likely to occupy the middle.

The choice of statistical model has a significant effect on the results, as well as their interpretation. A linear model would have been ideal since it would have provided a quantitative relationship between the predictors and the outcome. As this was not possible in the case of the HONC questionnaire results, ordinal, and logistic regression models were used instead, which allow for qualitative or semi-quantitative interpretations of the results. It is possible to speak about the relative strength of associations without being able to determine their nature (causal or confounding) or the absolute effect of the predictors on the outcome. This may explain the apparent discrepancies in the results, such as that the age at first cigarette was significantly associated with the outcome in the ordinal regression model but not in the binary logistic model. The ordinal HONC outcome contained different information to the binary HONC outcome. It is not clear whether the prevalence of LOA alone is sufficiently clinically meaningful or whether the degree of LOA conveys necessary additional information. The results of this study seem to support the latter conjecture.

It is important to find effective ways to prevent children from smoking and to identify the effective strategies for dealing with nicotine dependence and LOA, as this will mitigate the negative consequences at an individual and at a population level. Policy makers should take all above findings into consideration given that smoking initiation occurs at a very early age, thereby trapping young children into a vicious cycle of nicotine dependence with severe negative health trajectories for the rest of their lives.

### 4.2. Limitations

Limitations of this study include its cross-sectional design, which does not allow for causal inferences with regard to certain identified associations. Furthermore, approximately 82% of respondents who claimed to be current users of conventional cigarettes responded to the HONC questionnaire. This response rate is sufficiently high; however, it is not possible to dismiss the risk of selection bias since refusal to respond may have been related to smoking status and LOA. Moreover, it is possible that prevalence of nicotine product use was under-reported. Since the study relies on self-reported data, there may be recall bias or social desirability bias, with students being reluctant to admit their smoking or symptoms of nicotine dependence. Although it was not possible to investigate attrition caused by reluctance to admit smoking, it was possible to investigate attrition caused by smokers who were reluctant to admit symptoms of nicotine dependence. The results suggest that attrition did not occur at this level. If social desirability bias had affected results, we would probably have expected frequent smokers to be more reluctant to respond to HONC, but it was actually the less frequent smokers who did not respond as often. In addition, a small number of children in Greece aged up to 15 are not enrolled in school. Smoking prevalence is speculated to be higher among children who do not attend school. For this reason, it is expected that the true prevalence of smoking and of LOA symptoms among middle-school adolescents in Greece would be slightly higher than estimated. All in all, it may be that certain associations identified in this study are not representative of all Greek adolescents.

## 5. Conclusions

A high prevalence of nicotine dependence was found among adolescent Greek smokers. Those who smoke more frequently and those who started smoking at a younger age were found to have higher levels of LOA, although the direction of causality in the prior association is not clear. The prevalence of LOA was higher among girls, but the degree of LOA did not differ between genders. Differences in the age groups involved, in the types of nicotine products investigated, and in the statistical methods employed may account for certain differences between this and related studies, with regard to the relationship of gender and LOA.

## Figures and Tables

**Table 1:** Frequency analysis of population characteristics.

		Point Estimate	95% CI		*n*
HONC score categories	0 1–5 6–10	11.2% 56.5% 32.3%	7.84% 51.2% 27.3%	14.5% 61.8% 37.3%	339
Age	13 years 14 years 15 years	15.8% 37.6% 46.6%	11.9% 32.4% 41.3%	19.7% 42.7% 51.9%	339
Gender	Male Female	51.2% 48.8%	45.9% 43.5%	56.5% 54.1%	339
Cigarettes per day	≤5 cigarettes >5 cigarettes	70.7% 29.3%	65.8% 24.4%	75.6% 34.2%	329
Age at first cigarette	11 or younger 12 or older	26.7% 73.3%	22.0% 68.5%	31.5% 78.0%	334
Smoking in the family	No Yes	0.2% 99.8%	0.0% 99.3%	0.72% 100.0%	339

HONC: Hooked-on-Nicotine Checklist.

**Table 2:** Simple univariate analysis of the association between predictors and loss of autonomy outcomes

Predictors	Quantitative Outcome		Qualitative Outcome			
	Median	*p*-Value	Proportions			*p*-Value
	Score (0–10)		**0**	1–5	6–10	
Age 13 years 14 years 15 years	5 4 3.6	0.786	18.9% 8.7% 10.8%	39.6% 57.5% 61.4%	41.5% 33.9% 27.8%	0.053
Gender Male Female	4 4	0.425	15.5% 6.7%	50.0% 63.4%	34.5% 29.9%	0.11
Cigarettes per day ≤5 cigarettes >5 cigarettes	3 6	<0.001 *	14.2% 2.1%	63.1% 41.7%	22.7% 56.3%	<0.001 *
Age at 1st cigarette 11 or younger 12 or older	6 4	0.003 *	7.9% 12.7%	41.6% 61.6%	50.6% 25.7%	<0.001 *

* Significant *p*-values are denoted with an asterisk.

**Table 3:** Multiple univariate analysis of the association between predictors and loss of autonomy (HONC = 0 vs. 1–5 vs. 6–10)

Outcome:		Loss of Autonomy (LOA)		
Predictors		cOR	95% C.I.	*p*-Value
Age at first cigarette	11 or younger 12 or older	2.29 ref.	1.38–3.82	0.001 *
Cigarettes per day	≤5 cigarettes >5 cigarettes	0.240 ref.	0.144–0.400	<0.001 *

cOR: cumulative odds ratio. * Significant *p*-values are denoted with an asterisk.

**Table 4:** Investigation of bias due to attrition.

Characteristics		HONC Responders	HONC Non-Responders	*p*-Value
Age	13 years old 14 years old 15 years old	15.8% 37.6% 46.6%	28.0% 31.0% 41.0%	0.065
Gender	Male Female	51.2% 48.8%	59.1% 40.9%	0.251
Age at first cigarette	11 or younger 12 or older	26.7% 73.3%	26.6% 73.4%	0.985
Cigarettes per day	≤5 cigarettes >5 cigarettes	70.7% 29.3%	91.1% 8.9%	<0.001 *

* Significant *p*-values are denoted with an asterisk.

## Supplementary Materials

The following are available online at https://www.mdpi.com/1660-4601/17/21/8191/s1, File S1: The full database (Final database—Cig smokers.xls), File S2: The results of multiple binary logistic regression (Multiple logistic regression.doc).
