# Supplementary material for: Nicotine Dependence and Loss of Autonomy among Greek Adolescent Smokers: A Countrywide Cross-Sectional Study
Source: Int J Environ Res Public Health. 2020 Nov 5;17(21):8191. doi: 10.3390/ijerph17218191 (PMC7664176; doi:10.3390/ijerph17218191)
Supplement: Supplementary file 1 [file ijerph-17-08191-s001.zip › Multiple logistic regression.doc]

**RESULTS OF MULTIPLE LOGISTIC REGRESSION**

**Logistic Regression (initial model with all variables and interactions)**

| **Case Processing Summary** | | | |
| --- | --- | --- | --- |
| Unweighted Cases^a^ | | N | Percent |
| Selected Cases | Included in Analysis | 325 | 95,9 |
|  | Missing Cases | 14 | 4,1 |
|  | Total | 339 | 100,0 |
| Unselected Cases | | 0 | ,0 |
| Total | | 339 | 100,0 |
| a. If weight is in effect, see classification table for the total number of cases. | | | |

| **Dependent Variable Encoding** | |
| --- | --- |
| Original Value | Internal Value |
| 0 | 0 |
| 1 | 1 |

| **Categorical Variables Codings** | | | | |
| --- | --- | --- | --- | --- |
|  | | Frequency | Parameter coding | |
|  |  |  | (1) | (2) |
| Age | 13 years old | 50 | 1,000 | ,000 |
|  | 14 years old | 119 | ,000 | 1,000 |
|  | 15 yeas old | 156 | ,000 | ,000 |
| Cigs per day | 5 or less | 234 | 1,000 |  |
|  | 6 or more | 91 | ,000 |  |
| Age at first cig | 11 or younger | 79 | 1,000 |  |
|  | 12 or older | 246 | ,000 |  |
| Sex | Male | 157 | 1,000 |  |
|  | Female | 168 | ,000 |  |

**Block 0: Beginning Block**

| **Classification Table^a,b^** | | | | | |
| --- | --- | --- | --- | --- | --- |
|  | Observed | | Predicted | | |
|  |  |  | Dichotomous HONC Score | | Percentage Correct |
|  |  |  | 0 | 1 |  |
| Step 0 | Dichotomous HONC Score | 0 | 0 | 36 | ,0 |
|  |  | 1 | 0 | 289 | 100,0 |
|  | Overall Percentage | |  |  | 89,0 |
| a. Constant is included in the model. | | | | | |
| b. The cut value is ,500 | | | | | |

| **Variables in the Equation** | | | | | | | |
| --- | --- | --- | --- | --- | --- | --- | --- |
|  | | B | S.E. | Wald | df | Sig. | Exp(B) |
| Step 0 | Constant | 2,093 | ,177 | 139,182 | 1 | ,000 | 8,110 |

| **Variables not in the Equation** | | | | | |
| --- | --- | --- | --- | --- | --- |
|  | | | Score | df | Sig. |
| Step 0 | Variables | Age | 4,453 | 2 | ,108 |
|  |  | Age(1) | 4,444 | 1 | ,035 |
|  |  | Age(2) | ,657 | 1 | ,418 |
|  |  | Sex(1) | 5,056 | 1 | ,025 |
|  |  | Age at first cig(1) | 1,006 | 1 | ,316 |
|  |  | Cigs per day(1) | 9,452 | 1 | ,002 |
|  |  | Age * Cigs per day | 11,618 | 2 | ,003 |
|  |  | Age(1) by Cigs per day(1) | 10,656 | 1 | ,001 |
|  |  | Age(2) by Cigs per day(1) | ,043 | 1 | ,835 |
|  |  | Sex(1) by Cigs per day(1) | 14,864 | 1 | ,000 |
|  |  | Age at first cig(1) by Cigs per day(1) | ,173 | 1 | ,678 |
|  | Overall Statistics | | 24,040 | 9 | ,004 |

**Block 1: Method = Enter**

| **Omnibus Tests of Model Coefficients** | | | | |
| --- | --- | --- | --- | --- |
|  | | Chi-square | df | Sig. |
| Step 1 | Step | 24,037 | 9 | ,004 |
|  | Block | 24,037 | 9 | ,004 |
|  | Model | 24,037 | 9 | ,004 |

| **Model Summary** | | | |
| --- | --- | --- | --- |
| Step | -2 Log likelihood | Cox & Snell R Square | Nagelkerke R Square |
| 1 | 200,951^a^ | ,071 | ,143 |
| a. Estimation terminated at iteration number 20 because maximum iterations has been reached. Final solution cannot be found. | | | |

| **Hosmer and Lemeshow Test** | | | |
| --- | --- | --- | --- |
| Step | Chi-square | df | Sig. |
| 1 | ,955 | 7 | ,996 |

| **Contingency Table for Hosmer and Lemeshow Test** | | | | | | |
| --- | --- | --- | --- | --- | --- | --- |
|  | | Dichotomous HONC Score = 0 | | Dichotomous HONC Score = 1 | | Total |
|  |  | Observed | Expected | Observed | Expected |  |
| Step 1 | 1 | 6 | 7,007 | 14 | 13,353 | 20 |
|  | 2 | 8 | 7,368 | 32 | 32,867 | 40 |
|  | 3 | 9 | 8,279 | 38 | 38,503 | 47 |
|  | 4 | 4 | 3,434 | 21 | 21,618 | 25 |
|  | 5 | 2 | 3,630 | 44 | 42,953 | 47 |
|  | 6 | 3 | 2,526 | 31 | 31,402 | 34 |
|  | 7 | 2 | 1,770 | 34 | 33,888 | 36 |
|  | 8 | 1 | 1,040 | 32 | 31,859 | 33 |
|  | 9 | 1 | ,631 | 43 | 42,973 | 44 |

| **Classification Table^a^** | | | | | |
| --- | --- | --- | --- | --- | --- |
|  | Observed | | Predicted | | |
|  |  |  | Dichotomous HONC Score | | Percentage Correct |
|  |  |  | 0 | 1 |  |
| Step 1 | Dichotomous HONC Score | 0 | 0 | 36 | ,0 |
|  |  | 1 | 0 | 289 | 100,0 |
|  | Overall Percentage | |  |  | 89,0 |
| a. The cut value is ,500 | | | | | |

| **Variables in the Equation** | | | | | | | | | |
| --- | --- | --- | --- | --- | --- | --- | --- | --- | --- |
|  | | B | S.E. | Wald | df | Sig. | Exp(B) | 95% C.I.for EXP(B) | |
|  |  |  |  |  |  |  |  | Lower | Upper |
| Step 1^a^ | Age |  |  | ,066 | 2 | ,968 |  |  |  |
|  | Age(1) | 17,778 | 10622,503 | ,000 | 1 | ,999 | 52600692,778 | ,000 | . |
|  | Age(2) | ,403 | 1,568 | ,066 | 1 | ,797 | 1,496 | ,069 | 32,305 |
|  | Sex(1) | -,231 | 1,394 | ,027 | 1 | ,869 | ,794 | ,052 | 12,205 |
|  | Age at first cig(1) | ,269 | 1,584 | ,029 | 1 | ,865 | 1,309 | ,059 | 29,165 |
|  | Cigs per day(1) | -,912 | 1,160 | ,618 | 1 | ,432 | ,402 | ,041 | 3,902 |
|  | Age * Cigs per day |  |  | ,047 | 2 | ,977 |  |  |  |
|  | Age(1) by Cigs per day(1) | -18,726 | 10622,503 | ,000 | 1 | ,999 | ,000 | ,000 | . |
|  | Age(2) by Cigs per day(1) | -,353 | 1,632 | ,047 | 1 | ,829 | ,702 | ,029 | 17,211 |
|  | Sex(1) by Cigs per day(1) | -,745 | 1,452 | ,263 | 1 | ,608 | ,475 | ,028 | 8,174 |
|  | Age at first cig(1) by Cigs per day(1) | ,045 | 1,657 | ,001 | 1 | ,978 | 1,046 | ,041 | 26,919 |
|  | Constant | 3,383 | 1,088 | 9,677 | 1 | ,002 | 29,470 |  |  |
| a. Variable(s) entered on step 1: Age, Sex, Age at first cig, Cigs per day, Age * Cigs per day , Sex * Cigs per day , Age at first cig * Cigs per day . | | | | | | | | | |

**Logistic Regression (final model)**

| **Case Processing Summary** | | | |
| --- | --- | --- | --- |
| Unweighted Cases^a^ | | N | Percent |
| Selected Cases | Included in Analysis | 325 | 95,9 |
|  | Missing Cases | 14 | 4,1 |
|  | Total | 339 | 100,0 |
| Unselected Cases | | 0 | ,0 |
| Total | | 339 | 100,0 |
| a. If weight is in effect, see classification table for the total number of cases. | | | |

| **Dependent Variable Encoding** | |
| --- | --- |
| Original Value | Internal Value |
| 0 | 0 |
| 1 | 1 |

| **Categorical Variables Codings** | | | | |
| --- | --- | --- | --- | --- |
|  | | Frequency | Parameter coding | |
|  |  |  | (1) | (2) |
| Age | 13 years old | 50 | ,000 | ,000 |
|  | 14 years old | 119 | 1,000 | ,000 |
|  | 15 yeas old | 156 | ,000 | 1,000 |
| Cigs per day | 5 or less | 234 | ,000 |  |
|  | 6 or more | 91 | 1,000 |  |
| Age at first cig | 11 or younger | 79 | ,000 |  |
|  | 12 or older | 246 | 1,000 |  |
| Sex | Male | 157 | ,000 |  |
|  | Female | 168 | 1,000 |  |

**Block 0: Beginning Block**

| **Classification Table^a,b^** | | | | | |
| --- | --- | --- | --- | --- | --- |
|  | Observed | | Predicted | | |
|  |  |  | Dichotomous HONC Score | | Percentage Correct |
|  |  |  | 0 | 1 |  |
| Step 0 | Dichotomous HONC Score | 0 | 0 | 36 | ,0 |
|  |  | 1 | 0 | 289 | 100,0 |
|  | Overall Percentage | |  |  | 89,0 |
| a. Constant is included in the model. | | | | | |
| b. The cut value is ,500 | | | | | |

| **Variables in the Equation** | | | | | | | |
| --- | --- | --- | --- | --- | --- | --- | --- |
|  | | B | S.E. | Wald | df | Sig. | Exp(B) |
| Step 0 | Constant | 2,093 | ,177 | 139,182 | 1 | ,000 | 8,110 |

| **Variables not in the Equation** | | | | | |
| --- | --- | --- | --- | --- | --- |
|  | | | Score | df | Sig. |
| Step 0 | Variables | Age | 4,453 | 2 | ,108 |
|  |  | Age(1) | ,657 | 1 | ,418 |
|  |  | Age(2) | ,597 | 1 | ,440 |
|  |  | Sex(1) | 5,056 | 1 | ,025 |
|  |  | Age at first cig(1) | 1,006 | 1 | ,316 |
|  |  | Cigs per day(1) | 9,452 | 1 | ,002 |
|  | Overall Statistics | | 20,179 | 5 | ,001 |

**Block 1: Method = Enter**

| **Omnibus Tests of Model Coefficients** | | | | |
| --- | --- | --- | --- | --- |
|  | | Chi-square | df | Sig. |
| Step 1 | Step | 22,210 | 5 | ,000 |
|  | Block | 22,210 | 5 | ,000 |
|  | Model | 22,210 | 5 | ,000 |

| **Model Summary** | | | |
| --- | --- | --- | --- |
| Step | -2 Log likelihood | Cox & Snell R Square | Nagelkerke R Square |
| 1 | 202,778^a^ | ,066 | ,132 |
| a. Estimation terminated at iteration number 6 because parameter estimates changed by less than ,001. | | | |

| **Hosmer and Lemeshow Test** | | | |
| --- | --- | --- | --- |
| Step | Chi-square | df | Sig. |
| 1 | 1,571 | 7 | ,980 |

| **Contingency Table for Hosmer and Lemeshow Test** | | | | | | |
| --- | --- | --- | --- | --- | --- | --- |
|  | | Dichotomous HONC Score = 0 | | Dichotomous HONC Score = 1 | | Total |
|  |  | Observed | Expected | Observed | Expected |  |
| Step 1 | 1 | 6 | 6,548 | 14 | 13,813 | 20 |
|  | 2 | 8 | 7,604 | 32 | 32,631 | 40 |
|  | 3 | 5 | 5,352 | 25 | 25,221 | 31 |
|  | 4 | 7 | 5,905 | 33 | 33,328 | 39 |
|  | 5 | 3 | 4,181 | 46 | 44,430 | 49 |
|  | 6 | 3 | 2,619 | 31 | 31,308 | 34 |
|  | 7 | 1 | 1,510 | 24 | 23,868 | 25 |
|  | 8 | 1 | 1,076 | 33 | 32,110 | 33 |
|  | 9 | 2 | ,890 | 52 | 52,706 | 54 |

| **Classification Table^a^** | | | | | |
| --- | --- | --- | --- | --- | --- |
|  | Observed | | Predicted | | |
|  |  |  | Dichotomous HONC Score | | Percentage Correct |
|  |  |  | 0 | 1 |  |
| Step 1 | Dichotomous HONC Score | 0 | 0 | 36 | ,0 |
|  |  | 1 | 0 | 289 | 100,0 |
|  | Overall Percentage | |  |  | 89,0 |
| a. The cut value is ,500 | | | | | |

| **Variables in the Equation** | | | | | | | | | |
| --- | --- | --- | --- | --- | --- | --- | --- | --- | --- |
|  | | B | S.E. | Wald | df | Sig. | Exp(B) | 95% C.I.for EXP(B) | |
|  |  |  |  |  |  |  |  | Lower | Upper |
| Step 1^a^ | Age |  |  | 4,149 | 2 | ,126 |  |  |  |
|  | Age(1) | ,908 | ,486 | 3,497 | 1 | ,061 | 2,480 | ,957 | 6,426 |
|  | Age(2) | ,815 | ,466 | 3,062 | 1 | ,080 | 2,258 | ,907 | 5,624 |
|  | Sex(1) | ,931 | ,389 | 5,731 | 1 | ,017 | 2,536 | 1,184 | 5,433 |
|  | Age at first cig(1) | -,339 | ,464 | ,533 | 1 | ,465 | ,713 | ,287 | 1,770 |
|  | Cigs per day(1) | 1,847 | ,700 | 6,959 | 1 | ,008 | 6,340 | 1,608 | 25,008 |
|  | Constant | ,981 | ,524 | 3,504 | 1 | ,061 | 2,667 |  |  |
| a. Variable(s) entered on step 1: Age, Sex, Age at first cig, Cigs per day. | | | | | | | | | |
